# Data on alteration of hormone and growth factor receptor profiles over progressive passages of breast cancer cell lines representing different clinical subtypes

**DOI:** 10.1016/j.dib.2016.07.001

**Published:** 2016-07-06

**Authors:** Madhumathy G. Nair, Krisha Desai, Jyothi S. Prabhu, P.S. Hari, Jose Remacle, T.S. Sridhar

**Affiliations:** Division of Molecular Medicine, St. John׳s Research Institute, Bangalore, India

**Keywords:** Breast cancer, Cell lines, Immunofluorescence

## Abstract

Human breast cancers are a highly heterogeneous group of tumours consisting of several molecular subtypes with a variable profile of hormone, growth factor receptors and cytokeratins [1]. Here, the data shows immunofluorescence profiling of four different cell lines belonging to distinct clinical subtypes of breast cancer. Post revival, the cell lines were passaged in culture and immunophenotyping was done for ER, HER-2, AR and EGFR. Data for the markers from early passage (5th) through passages as late as 25 for the different cell lines is presented.

## Specifications Table

**Table:** 

Subject area	*Biology*
More specific subject area	*Cancer Biology*
Type of data	*Image*
How data was acquired	*Fluorescent Microscope (Olympus BX 50)*
Data format	*Raw*
Experimental factors	*Immunofluorescence on cells growing under standard growth conditions*
Experimental features	*Four cell lines were grown under standard growth conditions and immunofluorescence assay done across progressive passages in culture*
Data source location	*Bangalore, India*
Data accessibility	*Data is within this article*

**Value of the data**

• This data shows that the expression pattern of critical growth modulating receptors is dynamic and hence researchers need to confirm the status over progressive passages.
• This data provides evidence to the scientific community that gene expression data from cell lines needs to be cross verified with protein detection.
• The data highlights the importance of noting the passage number of breast cancer cell lines used for experimental testing of pharmacological agents.

## 1 Data

Immunofluorescence was performed for ER, HER-2, AR and EGFR on 4 different cell lines – MCF7; luminal cell line, MDA-MB-231; triple negative cell line, BT-474 and MDA-MB-453; HER-2 amplified cell lines. The data presented includes immunofluorescence images at an early passage (5/8th), an intermediate passage (15/16th) and a late passage (24th to 26th) for the four cell lines.

## 2 Experimental design, materials and methods

### 2.1 Cell culture

The cell line MCF7 was cultivated in DMEM-Hi glucose supplemented with 10% FBS. BT-474 and MDA-MB- 453 were grown in RPMI 1640 media. All three cell lines were incubated at 37 °C and 5% CO_2_ humidified atmosphere. MDA-MB-231 was cultivated in L-15 (Lebovitz medium) supplemented with 10% FBS and incubated at 37 °C without CO_2_ humidified atmosphere [2].

### 2.2 Mycoplasma testing

Mycoplasma testing was done by seeding the cells on glass coverslips and after 24 h, stained with Hoechst 33342 (0.1 mg/ml in PBS) for 10 min. Microscopic analysis was done to detect the presence of mycoplasma. All cell lines were routinely tested for mycoplasma and were found to be negative.

### 2.3 Immunofluorescence

**Fig. 1:**
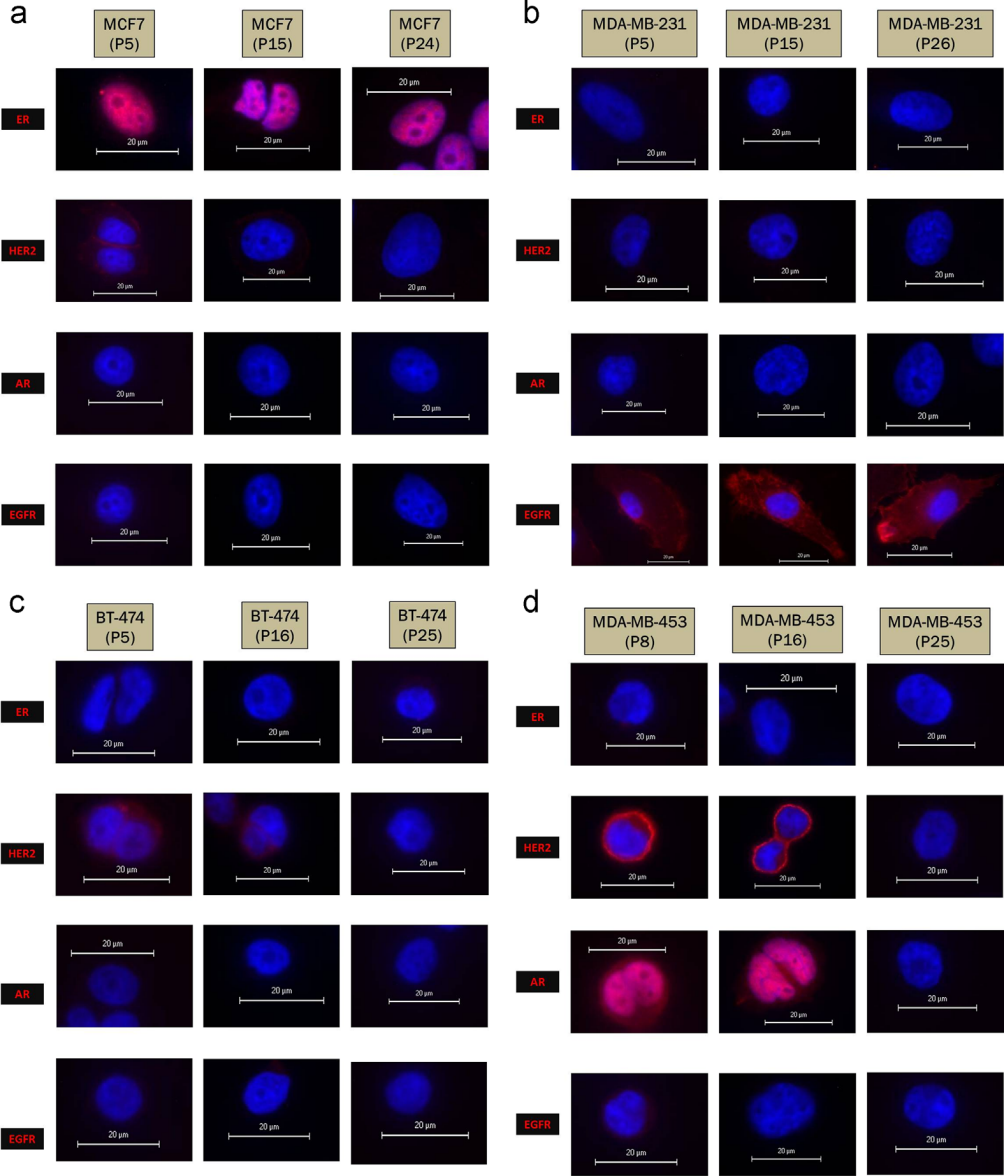
(a) MCF7 retains ER expression across all passages and has no expression of AR. There is mild expression of HER-2 and EGFR at passage 5 that becomes absent at later passages. (b) MDA-MB-231 shows absence of ER, HER-2 and AR across all passages and shows strong positivity for EGFR throughout. (c) BT-474 shows HER-2 positivity that becomes absent by passage 25. It shows no expression of ER, EGFR and AR. (d) MDA-MB-453 shows very strong HER-2 positivity that decreases considerably by passage 25. It shows no expression of ER but is mildly EGFR positive at passage 5. It is strongly AR positive as well which becomes absent by passage 25.

Immunofluorescence was done as described previously [2]. Briefly, cells were grown in 8 well slide chambers to 80% confluency. They were then fixed in 4% paraformaldehyde for 10 min. Permeabilisation and blocking was done with 2% FBS in 0.3% Triton X-100 (Calbiochem) in PBS followed by probing with the primary antibody and incubation overnight at 4 °C. The cells were then labelled with the corresponding secondary antibody Alexa Fluor 568 Donkey Anti-Rabbit IgG and AlexaFluor 568 Donkey Anti-Mouse IgG (H+L)(Invitrogen) for 1 h in a dark moist chamber. The slide was then mounted on gold antifade reagent with DAPI (Invitrogen) and examined under a fluorescent microscope (Olympus BX51). Anti-ER (Dako), anti-EGFR (Pathnsitu) and anti-AR (Pathnsitu) were ready-to use. Anti-HER-2 (Dako) was diluted 1:800 and secondary Alexafluor secondary antibodies were diluted 1:500 (Fig. 1).
