# Supplementary material for: Data on alteration of hormone and growth factor receptor profiles over progressive passages of breast cancer cell lines representing different clinical subtypes
Source: Data Brief. 2016 Jul 6;8:944–7. doi: 10.1016/j.dib.2016.07.001 (PMC4961495; doi:10.1016/j.dib.2016.07.001)
Supplement: Supplementary file 1 — Supplementary material [file mmc1.doc]

There are no potential conflicts of interest
